# Supplementary material for: meaRtools: An R package for the analysis of neuronal networks recorded on microelectrode arrays
Source: PLoS Comput Biol. 2018 Oct 1;14(10):e1006506. doi: 10.1371/journal.pcbi.1006506 (PMC6181426; doi:10.1371/journal.pcbi.1006506)
Supplement: S1 Text — (DOCX) [file pcbi.1006506.s001.docx]

S1 Text. Supplementary Methods

Mice

*Celf4*knockout mice on the 129S1/SvImJ inbred strain were used for these studies[1].  Homozygous and wildtype littermate pups used in MEA were obtained from crosses between heterozygotes.

Dissociation and culturing of primary mouse cortical neurons

Primary mouse cortical neurons were obtained from the new born P0-1 mice from the same litter. 48-well MEA plates were coated with 0.5 % PEI and laminin (20 ug/mL) prior to the seeding of disassociated neurons. Dissection and dissociation protocols were adopted and optimized from the published work of cortical culture on MEA [2]. Cerebral Cortex was removed in ice-cold Hank's Balanced Salt Solution (HBSS) buffer, mechanically disassociated, and subsequently enzymatically dissociated with Trypsin (8 minutes) followed by DNase treatment (6 minutes) at 37°C. Trypsin was neutralized by MEM (10% FBS, glucose, 5 mM HEPES, and Penicillin Streptomycin) and the suspension was centrifuged at 200 rcf for 5 min followed by trituration by flame-polished glass Pasteur pipette. 150,000 cells were seeded into each pre-coated well. Plating was done in a randomized pattern to control for potential confounding spatial effects on the MEA plate. MEM (10% FBS, glucose, 5 mM HEPES, and Penicillin Streptomycin) was replaced by Neurobasal-A media (Life Technologies) supplied with B-27, 5mM HEPES, and Penicillin Streptomycin after 3 h. Arabinofuranosyl cytidine (ara-C) (5 µM) was added to the cultured neurons at DIV3 and washed away at DIV5. 45% of media was changed every two days starting from DIV12.

References

1. Wagnon JL, Mahaffey CL, Sun W, Yang Y, Chao HT, Frankel WN. Etiology of a genetically complex seizure disorder in Celf4 mutant mice. Genes Brain Behav. 2011;10(7):765-77. doi: 10.1111/j.1601-183X.2011.00717.x. PubMed PMID: 21745337; PubMed Central PMCID: PMCPMC3190060.

2. Valdivia P, Martin M, LeFew WR, Ross J, Houck KA, Shafer TJ. Multi-well microelectrode array recordings detect neuroactivity of ToxCast compounds. Neurotoxicology. 2014;44:204-17. doi: 10.1016/j.neuro.2014.06.012. PubMed PMID: 24997244.
